# maxdLoad2 and maxdBrowse: standards-compliant tools for microarray experimental annotation, data management and dissemination

**DOI:** 10.1186/1471-2105-6-264

**Published:** 2005-11-03

**Authors:** David Hancock, Michael Wilson, Giles Velarde, Norman Morrison, Andrew Hayes, Helen Hulme, A Joseph Wood, Karim Nashar, Douglas B Kell, Andy Brass

**Affiliations:** 1 School of Computer Science, The University of Manchester, Kilburn Building, Oxford Road, Manchester, UK; 2 School of Chemistry, The University of Manchester, Faraday Building, PO Box 88, Sackville Street, Manchester, UK; 3 Faculty of Life Sciences, The University of Manchester, Oxford Road, Manchester, UK; 4 NERC Environmental Bioinformatics Centre, Oxford Centre for Ecology and Hydrology, Oxford, UK

## Abstract

**Background:**

maxdLoad2 is a relational database schema and Java^® ^application for microarray experimental annotation and storage. It is compliant with all standards for microarray meta-data capture; including the specification of what data should be recorded, extensive use of standard ontologies and support for data exchange formats. The output from maxdLoad2 is of a form acceptable for submission to the ArrayExpress microarray repository at the European Bioinformatics Institute. maxdBrowse is a PHP web-application that makes contents of maxdLoad2 databases accessible via web-browser, the command-line and web-service environments. It thus acts as both a dissemination and data-mining tool.

**Results:**

maxdLoad2 presents an easy-to-use interface to an underlying relational database and provides a full complement of facilities for browsing, searching and editing. There is a tree-based visualization of data connectivity and the ability to explore the links between any pair of data elements, irrespective of how many intermediate links lie between them. Its principle novel features are:

• the flexibility of the meta-data that can be captured,

• the tools provided for importing data from spreadsheets and other tabular representations,

• the tools provided for the automatic creation of structured documents,

• the ability to browse and access the data via web and web-services interfaces.

Within maxdLoad2 it is very straightforward to customise the meta-data that is being captured or change the definitions of the meta-data. These meta-data definitions are stored within the database itself allowing client software to connect properly to a modified database without having to be specially configured. The meta-data definitions (configuration file) can also be centralized allowing changes made in response to revisions of standards or terminologies to be propagated to clients without user intervention.

maxdBrowse is hosted on a web-server and presents multiple interfaces to the contents of maxd databases. maxdBrowse emulates many of the browse and search features available in the maxdLoad2 application via a web-browser. This allows users who are not familiar with maxdLoad2 to browse and export microarray data from the database for their own analysis. The same browse and search features are also available via command-line and SOAP server interfaces. This both enables scripting of data export for use embedded in data repositories and analysis environments, and allows access to the maxd databases via web-service architectures.

**Conclusion:**

maxdLoad2  and maxdBrowse  are portable and compatible with all common operating systems and major database servers. They provide a powerful, flexible package for annotation of microarray experiments and a convenient dissemination environment. They are available for download and open sourced under the Artistic License.

## Background

Rich and accurate experimental annotation is important to the analysis and understanding of experimental results. This is especially true amongst the post-genomic techniques such as transcriptomics where, due to the scale of data produced, relatively minor changes in laboratory procedure can have profound effects on results and their interpretation. For this reason it is crucial that all relevant protocols and parameters, i.e. the experimental meta-data, are recorded.

The microarray community has agreed standards regarding both the data that should be captured (MIAME, [1]) and how these data should be modelled and exchanged (MAGE-OM [2], MAGE-ML, [3]). However, these standards are still evolving. Similarly, post-genomic technologies are continuing to evolve and are being applied to news areas, for example in environmental genomics and toxicogenomics. These new applications have very different data capture needs to those encountered in more standard model organisms. Indeed, simply using arrays with different classes of organisms can add to the complexity of the annotation required.

Ensuring compliance with standards means that the meta-data capture step should not be seen as an insurmountable barrier to progress. The standards for meta-data capture and exchange are still not readily usable or even understood by much of the biological community (for example the level of detail required for MIAME compliance [1] and the complexity of models used to store microarray data). A consequence of this complexity is that the model [2] can be seen to be confusing by bench biologists, a principal target audience of experimental annotation software. In addition, the associated MGED ontology [4] that provides controlled terms for annotation is continually evolving to cover new biological fields, new applications of the technology and to correct oversights. This must be reflected in the software so that the user can annotate their experiment with the fullest set of terms available.

The volume of data needing to be captured is growing rapidly. This increase in volume occurs in the numbers of experiments and replicates performed, as well as in the numbers of spots on each array. The increasing level of interest is due to more people becoming involved in transcriptomics research, whether they are collaborators or clients, or independent researchers browsing public data. Both of these factors necessitate software that can handle large amounts of data and present it to the end user in the most efficient way possible.

To meet these challenges, we believe that tools for annotation of transcriptomics experiments must offer four key features:

• flexibility, to adapt to changing standards and technologies;

• scalability, to cope with the increasing amounts of production and use of data;

• usability, to reflect the way users consider experiments and handle their data (including provision of an simplified view of the underlying data model at a level of abstraction suitable for biologists);

• distributability, to allow users to disseminate their data to colleagues and to public repositories in standards-compliant format for publication.

There are already a number of free software tools available to the community. MADAM: MicroArray Data Manager [5] provides a Java data entry tool over a relational database. BASE [6], Longhorn Array Database [7] and MARS [8] provide data capture and browsing tools through a Web server. MIAMExpress [9] provides web tools for entering data directly into ArrayExpress.

However, although each of these software tools are MIAME supportive and generally capable of providing MAGE-ML output, they did not meet the specific requirements we had identified for the creation of customised attributes to meet the needs of specific communities, support for bulk loading data from spreadsheets, or the ability to produce MAGE-ML that was sufficiently expressive to cope with situations in which users wished to customise their meta-data and meet the standards of repositories such as ArrayExpress. We summarise these tools and their capabilities in table 1.

**Table 1:** A comparison of the different products available to handle transcriptome experimental data

Software	Platforms	MIAME Compliant	Imports	Exports	Fully customisable attributes	Comments
maxdLoad2	Any that runs Java	Yes	maxd-ML, MAGE-ML, bulk loading from CSV files using XML	maxd-ML, text and fully expressive MAGE-ML	Yes	
maxdBrowse	Server requires Apache and PHP5. Clients any web browser or program/script that can be configured to retrieve data via SOAP (if remote) or from the command-line (if run on the server)	Same as maxdLoad2	No import	Exports plain-text, HTML and XML	Yes – runs off maxdLoad2 attributes	
MIAMExpress	Any (web-based)	Yes	Web-based forms	N/A	No	No bulk load facility, although it is being extended to allow uploading from spreadsheets in the future
BASE	Server is installed on GNU/Linux, with a web-based front-end	Optional	Web-based forms, bulk loading for raw data only	MAGE-ML	Potentially, via user definable fields	Plug-ins available for data normalisation, analysis and viewing
MADAM	MS Windows and GNU/Linux	Yes	Web-based forms, no bulk loading	Its own format (.mev)	Potentially, by editing the database schema	Is being adapted to read and write MAGE-ML in the future
LAD	Server is installed on GNU/Linux, with a web based front end	Yes	Batch imports using tab delimited files	Tab delimited file	No	Very limited experimental annotation. Open source version of the Stanford Microarray Database

Here we present two components of the maxd software suite, maxdLoad2 and maxdBrowse. The former is a robust, fully featured database management, annotation and export tool, available as a downloadable application. The latter is a dissemination and data-mining tool, available as a server-side application presenting browser-based, command-line and web-service interfaces to the data.

## Implementation

In contrast to many other transcriptomics database solutions, maxdLoad2 is a standalone application rather than a web-based tool. This allows a higher degree of interactivity whilst mitigating the considerable cost of data upload from browser to server. It is written in the Java programming language making it highly platform independent. The database back-end can be provided by any relational database server which implements the SQL92 standard, including Oracle, PostgreSQL and MySQL. The server can be accessed remotely, or for maximum performance can be run on the same machine as maxdLoad2. Whilst standalone application installation is rarely as simple as accessing a web based service, considerable effort has been put into making maxdLoad2 easy to get started. In the majority of cases installation is a matter of downloading and running a single file. The maxdLoad2 website contains full instructions on installation and configuration of Java as well as an appropriate SQL server product. In addition several commonly used microarray chip descriptions are available for download.

The ability to disseminate data stored in maxdLoad2 databases via a browser is important. Our solution was to develop one independently in a language built for this environment, PHP, which is also platform independent. Being a web-application, it is less easy to install, but once installed very easy to use on a browser.

Bioinformatics data-analysis demands keep on changing, but access to data and meta-data in a scriptable form is a must. maxdBrowse thus provides quick access to the contents of maxdLoad2 databases via the command-line and as a web-service.

### User interface, navigation and browsing

**Figure 1:**
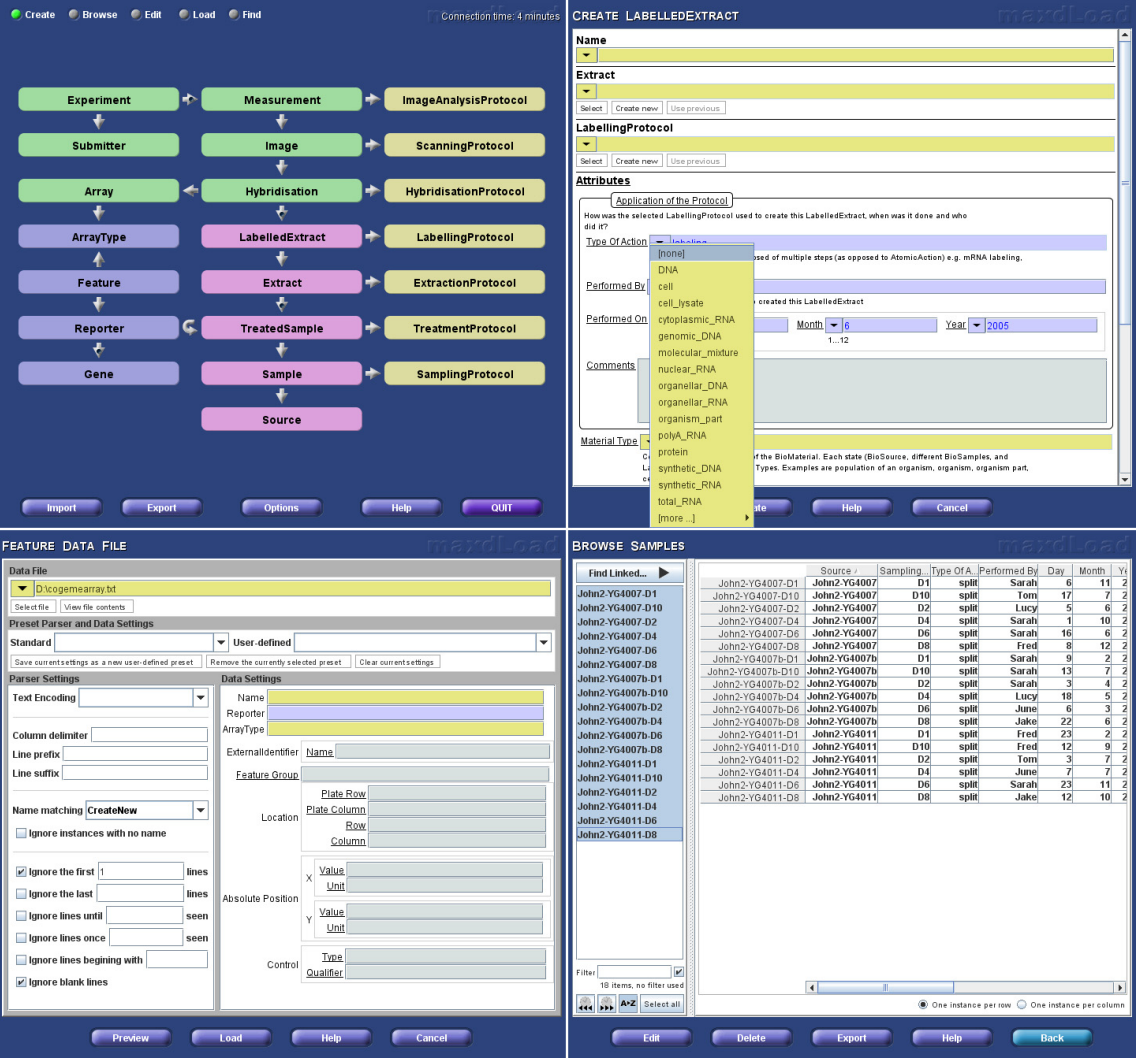
**The maxdLoad2 interface**. Clockwise from the top-left, this figure shows (1) the main schema interface window (2) an example of a create mode manual entry form, (3) Browse Mode example, for viewing instances in the database, (4) the Load Mode interface for populating tables from spreadsheets.

The complexity of the schema, simple as it is compared to the full MAGE-OM, still represents a challenge for visualisation and navigation. A graphical representation of the schema appears at the top-level of the user interface and is used as the principal navigation tool (figure 1(a)). Users click on elements of the schema to jump directly to data entry or browsing forms for that element. The representation also attempts to make clear the relationship between schema elements. An optional alternative visualisation is a version of the schema flattened into a tree rooted at the Experiment table. Although table-interconnectivity is harder to portray in the tree paradigm, it has the advantage of being more readily able to display information about specific entries. An interactive highlighting scheme which displays the linkage of individual items as the mouse passes over them further enhances the usefulness of this representation.

maxdLoad2 has five principle interaction modes, 'Create', 'Browse', 'Load', 'Edit' and 'Find'. Each uses the same top-level schema-based navigation method to select particular database elements for manipulation.

Create mode displays blank forms, built from the XML specification for the table, to allow for annotation of a single entry (figure 1(b)). The data-types specified translate into standard interface elements such as free text entry boxes and drop-down menus. The Browse, Edit and Find modes allow interaction with existing entries (figure 1(c)). Navigation between entries is facilitated by the 'Find Linked' function which allows interconnected entries to be identified regardless of the underlying schema and the number of links between them. It finds the shortest path between the tables and issues the relevant series of queries to determine which entries are related. An example of this is identifying all 'Experiment' instances that use a particular 'TreatmentProtocol'.

**Figure 2:**
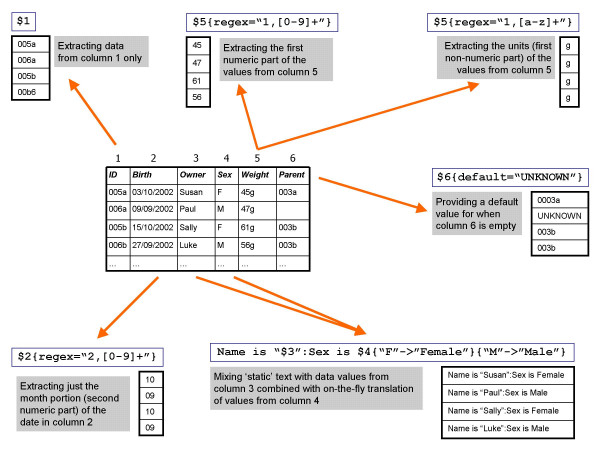
**Column specification Syntax for data loading**. The column specification syntax controls the destination attribute field that a column of data should be entered into in the database. The data can be pre-processed before they are loaded into the fields including data enumerations to convert values, combinations to allow multiple fields to be recomposed into an attribute and regular expressions to perform complex operations such as separating values from units, or removing punctuation, amongst other procedures.

As large amounts of microarray data and meta-data are in the form of tab-delimited or spreadsheet files, for example array descriptions, genome information and raw or processed data files, maxdLoad2 has specific features for handling such data. Load mode uses the familiar graphical interface of Create mode (figure 1(d)) but instead of providing values one-by-one, a flexible data description language is used to specify how to extract data from a file, as can be seen in figure 2. Various data manipulation tools are available including on-the-fly translation and regular expression pattern matching. Once the extraction rules have been specified, the data that will be entered into the database can be previewed by a built-in spreadsheet interface before loading.

**Figure 3:**
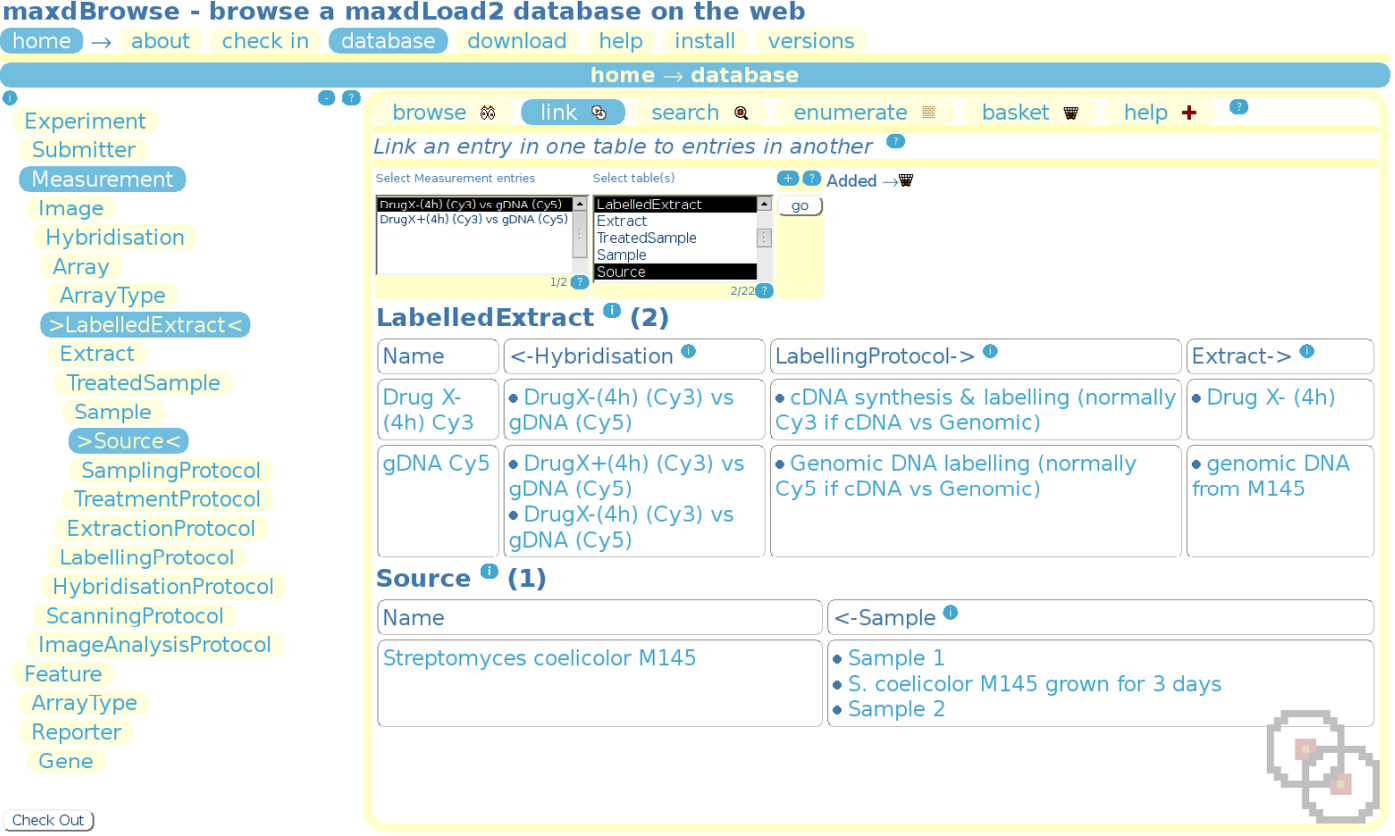
**The maxdBrowse interface**. This shows maxdBrowse linking an entry on the 'Measurement' table to entries in the 'LabelledExtract' and 'Source' tables. At the very top are site navigation tabs and a banner indicating that the database page is being accessed. Underneath this are tabs to switch between navigation modes, with link highlighted as the current mode. Underneath this is a selection form, where entries can be selected for linking to other tables, additional formatting options are available by clicking the (+) link on top of the 'go' button. Below this is the data presentation form showing the results of the query, in this case the 'Measurement' *DrugX-(4 h) (Cy3) vs gDNA (Cy5) *has been linked down to entries in LabelledExtract and Source. On the right is the TableNavigatorTree, highlighting the tables that are being linked. Help links (?) pop-up help about maxdBrowse features, and information links (i) pop-up help about maxdLoad2 schema elements displayed on the page.

maxdBrowse presents multiple interfaces to maxd databases built by maxdLoad2. Its web interface emulates the browsing capabilities of maxdLoad2 (figure 3), with a simpler table navigator, entry selection and various formatting options. maxdBrowse also provides a command-line SOAP server interface.

Several kinds of query are available, and these are reflected by links at the top to (currently) 'browse', 'link', 'search', 'enumerate' and 'basket'. Each of these modes gives a customised form for that kind of query. The primary query, used to navigate and inspect database, is 'browse'. Selected entries will be retrieved and formatted, displaying links to other parent and child entries in the schema. These links can be used to navigate through the schema to discover aspects of the experiment currently being browsed and various formatting options are available. It also allows the creation of simple structured documents, via use of the recursive 'View descendant' option, which trawls down the schema retrieving a selected entry's children, and those children's children etc.

The link query allows a selected entry in one table to be linked to entries in another, provided there is a direct path between the two tables in the schema, or a path can be made across ArrayType (which is linked to by Array up to Experiment on one side, and by Feature down to Gene on the other). The search query allows searching for entries in either one or all tables. There is a basic search, allowing a quick search of entry names or all the attributes for each entry, and an advanced search, where individual database fields can be searched. Advanced search provides the ability to search ontologies used in the database, including the standard MGED ontology classes used in the maxdLoad2 schema. The web interface provides ontology term selection forms for these. This allows relations between entries to be explored using browse and link-modes, and entry attributes to be searched using free-text and fixed-term strings in search-mode. The enumerate query allows retrieval of numerical data; when run it initially provides column meta-data for selected Measurements, allowing the user to identify the columns of interest for retrieval. The user is then given the option of linking the results to the relevant Feature, Reporter and Gene attributes, and also to select a subset of genes for which to return results.

**Figure 4:**
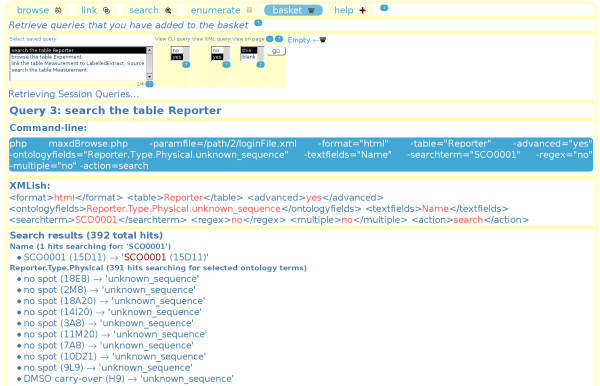
**The maxdBrowse query syntax**. This shows maxdBrowse in basket-mode, retrieving one of several queries previously performed and saved to basket. The query shown is an advanced search of the Reporter table, where the Type. Physical ontology field is being searched for 'unknown_sequence' and the 'Name' free-text field is being searched for 'SCO001'. Also shown are examples of the command-line and XML query syntax that can be used to generate the same result

Each query can be saved to the basket, and retrieved at a later time. This allows the user the opportunity to build a report of useful meta-data, in much the same way online-shoppers select items that they wish to buy. The basket has an option to display command-line and XML format query syntax (see figure 4). Thus if the user finds that reports are getting large, or if the same kinds of reports are being repeatedly generated, then by providing these examples maxdBrowse shows the user how to perform them in command-line syntax. Programmers who wish to use maxdBrowse as an API can try out example queries 'by hand' on the web-page first, and then retrieve them in the basket for use in their SOAP client/command-line code.

### Data storage

**Figure 5:**
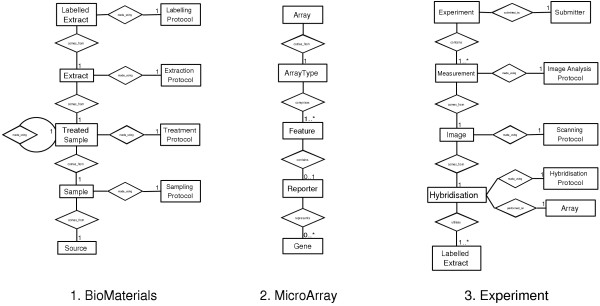
**Entity relationship diagram**. This shows the relationships between the tables in the maxd database schema.

**Figure 6:**
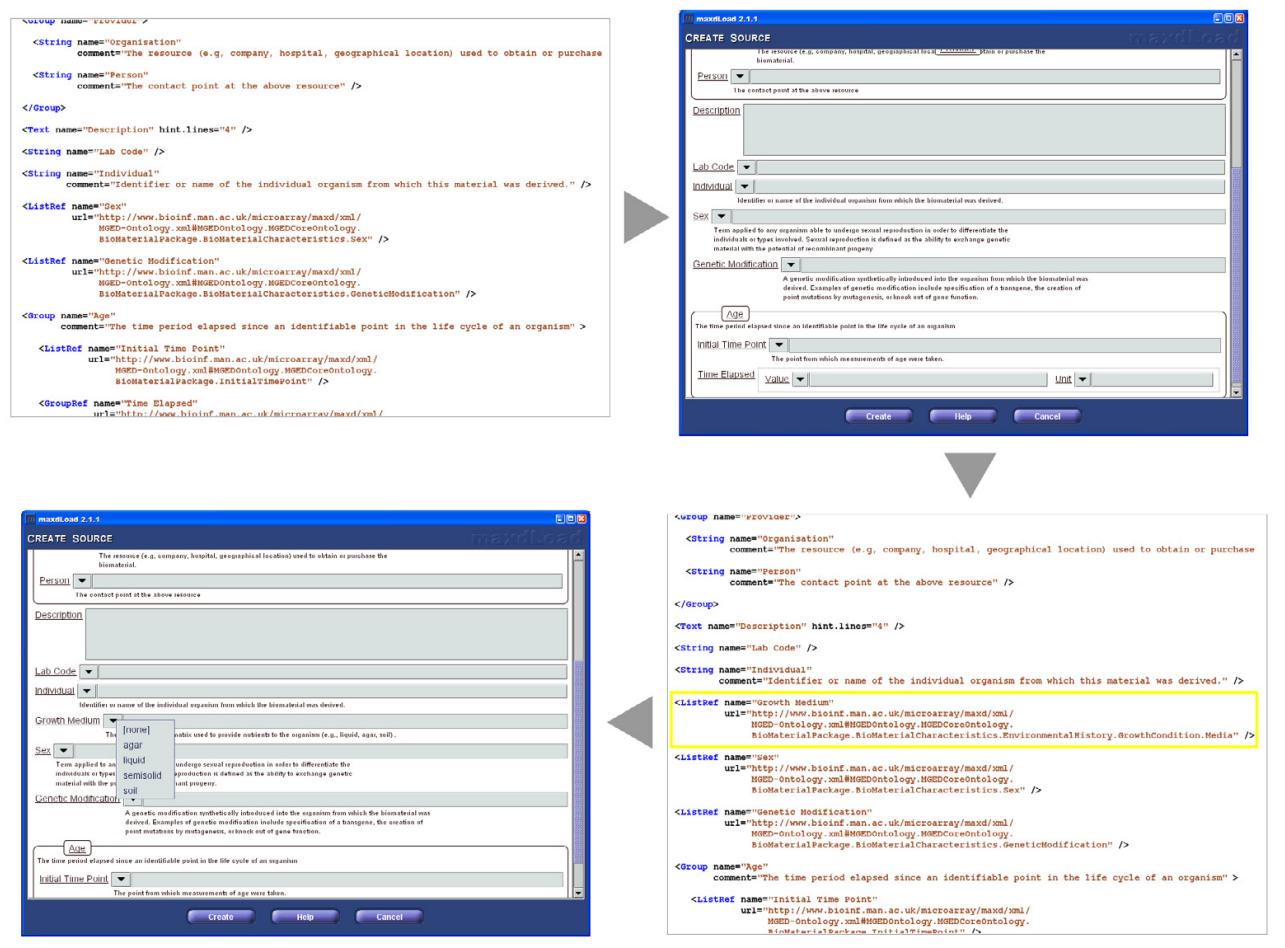
**Attribute Definition Mechanism**. Clockwise from the top-left, this figure shows (1) a portion of the standard attribute definition file, (2) the section of the user interface that is generated from this definition, (3) customising the attribute definition by adding a new item "Growth Medium" and (4) how the user interface changes in response to the change.

The schema is loosely based on the ArrayExpress database model [10] and it maps onto the MAGE-OM [12,3], with modifications for reasons of simplicity, efficiency and future configurability. The maxd schema is made up of relational and xml-specified components. The relational component covers the general more static table-structure (figure 5), representing both the links between and names of entries in various tables. The dynamic meta-data is stored in attribute columns in a more flexible structure of xml-defined name/value pairs. The attribute specifications are stored in a file which defines the collection of meta-data to be stored for each table in the database. The standard attribute definition is built from the MIAME specifications and MGED ontology, but this can be easily customised from directly within maxdLoad2. This allows attributes to be modified to any degree without requiring a change to the underlying database structure, allowing the software to adapt to changes in details of the model; this is seen in figure 6.

### Data import and export

Other than the form-based annotation and loading maxdLoad2 has bulk export and import facilities to assist in administration and data sharing. These include a native XML based format for transferring data between databases and disseminating complex items such as array descriptions to users. In addition there is a tab-delimited export mode for database entries, a tabular export option for measurement data and the ability to generate structured documents, including, but not limited to, MAGE-ML, via a template based export schema.

Programmatic access to the database is possible via a library API which exposes much of maxdLoad2's functionality to other applications capable of interacting with Java methods.

maxdBrowse opens up maxd databases to web dissemination, and provides additional export facilities, including tab-delimited, flat text, HTML and XML output, while a recursive search over links between entries allows for experimental report generation. The use of maxdBrowse on the command-line and in scripts allows the contents of maxd databases to be made available to a variety of tools and interfaces, for example R/Bioconductor [11] or MATLAB. maxdBrowse is also accessible via SOAP, allowing its extension as a web service to clients across the internet. Example SOAP client interfaces, initially in Perl and PHP, are included with the distribution to help those who wish to build their own interfaces, and web-services description by WSDL is also in progress.

### Usability

The software has undergone several rounds of cyclical testing and modification in usability studies with lab biologists using standard cooperative evaluation methodologies [12]. The results of one of these studies are freely available [13]. This has led to several major enhancements to the software's ease of use and applicability to the process.

## Results & discussion

**Figure 7:**
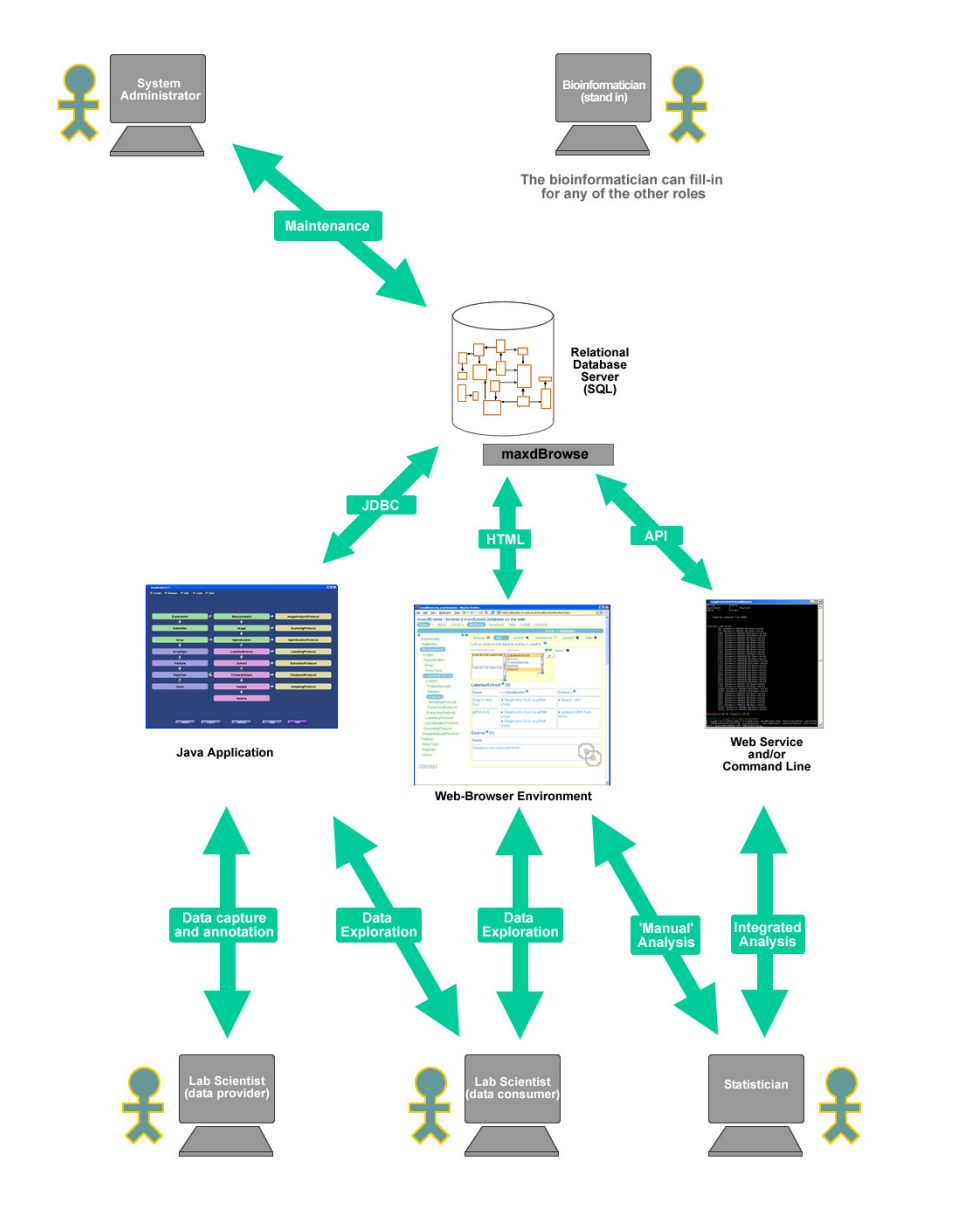
**A Use-case Diagram for maxdLoad2 and maxdBrowse**. The 'system administrator' is the individual in charge of maintaining the lab's database and web server. The 'lab scientist (data provider)' is typically someone who does a lot of microarray experiments and manages his experiments using maxdLoad2. The 'lab scientist (data consumer)' is typically someone who may not perform as many microarray experiments, and may not even work in a microarray facility, but is interested in the data and could use maxdBrowse or maxdLoad2 for its access. The statistician would be interested in retrieving data and meta-data for analysis, and may prefer to access the data programmatically either with scripts using maxdBrowse on the command-line or via its SOAP interface. The 'bioinformatician (stand-in)' may be involved at any level with any of these activities.

The requirements for flexibility and usability could be seen as mutually exclusive. It is typically the case that software which is very configurable requires effort to set up and use. Conversely, software which is very easy to use typically achieves this by restricting flexibility. The maxd suite of software is therefore aimed at three specific user groups, summarised in figure 7.

Deciding what meta-data should be captured for a particular series of experiments and then capturing it effectively in MAGE-ML requires a user group with considerable experience – typically a bioinformatician working with the bench biologists. maxdLoad2 provides such users with a variety of tools for configuring the interface and the MAGE-ML output relatively straightforwardly. If a lot of the meta-data has already been captured in the form of spreadsheets they can also configure the software to take data directly from them.

Once the database and interfaces developed by the bioinformaticians have been set up, they are relatively straightforward to use by the bench biologists (as evidenced from the usability studies). A useful feature is the way in which the data can be provided to the software in the form of spreadsheets. In practice we have seen that this task is also often undertaken, not by the bench biologists, but by a bioinformatician responsible for capturing all the meta-data from experiments run within a particular facility. maxdLoad2 provides good tools for allowing efficient bulk loading of meta-data to help with this. This is also typically the point at which data are exported from maxdLoad2 into ArrayExpress.

Once the data have been captured within the database there is then a requirement to allow co-workers or collaborators to readily explore which experiments have been run and download some of the data if required. These users will probably not want to learn maxdLoad2 and their needs are met by maxdBrowse.

A final set of users that we should be considering are software programs wishing to access array data for use in larger, integrated genomics studies. These needs could potentially be met through the command-line interfaces to maxdBrowse and will be considerably expanded in the future.

### Complex models

Annotation of transcriptomics experiments is a hard problem, partly owing to the large amounts of both data and annotation and also to the complexity of the underlying model, which does not necessarily reflect the thinking of a lab biologist. For this reason the maxd suite has been designed with usability by laboratory experimentalists in mind. The first concession to this is that the maxdLoad2 database model is not based on the MAGE-OM itself, but an abstraction of the model, hiding much of the complexity, and addressing itself to how users represent their experiments, whilst still maintaining the overall structure of the model.

Separating the functionality of the standalone-application from the web-application is also crucial, by targeting different groups of users who vary in their ability to invest time and effort into learning new tools. maxdLoad2 and maxdBrowse form a synergistic suite, with maxdLoad2 being a very powerful and flexible tool for annotation and management of experiments, whilst maxdBrowse is a powerful tool for dissemination of experiment data sets, in an easy-access and easy-to-learn web browser based environment which avoids the need for collaborators or facility clients from having to learn to use the full standalone tool.

The large amount of data available from microarray experiments as well as the increasing numbers of people generating and using the data provides challenges for transcriptomics annotation software. The maxd suite provides several features for day-to-day users to ease use of the software. maxd databases can be populated with all standard laboratory protocols and arrays, freeing the end-user of this task and ensuring consistency and completeness of annotation within laboratories. All annotation and data can be bulk-loaded from Excel spreadsheets and other tab-delimited formats into maxdLoad2 databases with ease.

The maxdBrowse web-interface allows collaborators or clients to review annotated experiments in a simple manner at any time, without needing maxdLoad2. Its recursive browse ('View descendant') and basket functions help users create reports in a flexible manner. When run as a command-line tool maxdBrowse is capable of extracting large amounts of data from a maxd database. For instance, it is quite easy to write web pages that send requests from users to maxdBrowse and return their results by e-mail. Alternatively maxdBrowse's recursive browse function enables structured representations of an experiment to be created as an overview with associated annotation and data, though in a more restricted form than maxdLoad2's structured document generation. The advantage of doing this however, is the potential for scripting a range of different export queries into analysis environments, including R/Bioconductor and MATLAB. maxdBrowse also offers the potential for exposing the underlying functionality as a web-service. An example of a useful set of maxdBrowse scripted queries would be to use the link mode (as in figure 3, but not via the GUI) to identify each Measurement's HybridisationProtocol across all experiments in a database, and reclassify them based on this. The enumerate function could then retrieve the data from the 'Flags' column for each of these Measurements, and quickly work out the percentage failure rate, to determine if correlations can be made based on the protocol used. This could also be rapidly encapsulated in a PERL script to automate the process.

The maxd suite has been designed from the ground up to be highly flexible, allowing administrators to tailor the software for the particular needs of users. For example, the set of attributes associated with the database can be altered to suit the biological domain being annotated a feature that has been exploited in the development of MIAME/Env [14]; an extended MIAME specification where new attributes have been added to support annotation of microarray experiments carried out by the NERC Environmental Genomics community [15]. This is achieved by editing the XML-based attribute definition files using an editor built into the application. This mechanism also allows the software to adapt to changes in annotation standards as the definition of the standard terms is held on a globally visible central server which is updated to track changes in both the MIAME specification and the MGED Ontology. This centralized administration stretches to other areas of the software, such as Unicode definition files to change the range of symbols that can be included in annotation description, and to loading preset files, which control maxdLoad2's parser settings for loading data from spreadsheet files. These repositories are being filled with settings for both array layout and measurement files for the broad majority of independent manufacturers over time and can be added to to cater for custom laboratory formats.

The maxd software suite also offers flexibility in the sense that it enables data-producing centres to quickly establish their own microarray databases and publish them with web-based front-ends. This allows users and clients to interact with central repositories in a far more integrated and organized manner than sending raw scanner output files with an experimental description in an e-mail. Examples of web-based repositories that embed maxdBrowse for access to transcriptomics data include StreptoBASE 16 at the University of Manchester and EnvBrowse 17 at the Natural Environment Research Council (NERC) Environmental Bioinformatics Centre.

Capturing transcriptomics data is only the first part of the process; to be useful they need to be distributed to collaborators or to public repositories. Distribution of data is complicated by the varied backgrounds of individuals involved, the size of the data sets and the distributed nature of the resources. Web-based applications tend to be easy to use, but can only present a limited subset of data to the user, due to the speed of connection to the server and issues surrounding the capabilities of web clients. On the other hand, standalone applications tend to be more complicated to install and use, but are far more robust and powerful in the specific tasks they can perform. The maxd suite has been designed to maximize the advantages that these platform decisions offer, for example data entry can only be done in the standalone application, as this is a far better environment with instantaneous interface and high throughput data handling that experimental data loading requires. Data dissemination on the other hand can be performed in either maxdLoad2 or maxdBrowse, depending on the audience. Users who are comfortable with maxdLoad2 and trusted with the data can use it to browse the contents of databases, whereas other users can be given access to the database using maxdBrowse. Both components can perform data export; maxdLoad2 can export whole experiments, and any part thereof, whereas maxdBrowse focuses only on exporting selected entries and their descendants because of web-browser and service limitations (e.g., if an Experiment and 'View descendant' are selected, everything but array design would be exported).

By ensuring that the solutions are flexible, scalable and usable, organizing efficient data distribution networks becomes far more tractable. We believe maxdLoad2 is the application of choice for communication within and between data-centres, whether it be annotating experiments or exporting structured documents such as MAGE-ML. To this end, the European Bioinformatics Institute accepts maxdLoad2-generated MAGE-ML submissions into ArrayExpress. maxdBrowse, however, can act as a general dissemination medium for non-experts, by providing a simple web-interface for selecting, retrieving and formatting the contents of maxd databases. On the command-line, its ability to query maxd databases with a simple set of parameters allows mining of these data by a variety of possible interfaces and analysis solutions, which may or may not be located at the same site as the database itself.

### Planned future development

Currently, the maxdLoad2 security model is based on the rather coarse-grained security provided by the default underlying database server. This implies that a user with sufficient database privileges could perform actions on any data that they can see. Due to the lack of "row-level" security, it is not possible to control access to a subset of a particular database. Our interim solution is to advise users to disseminate data using the maxdBrowse web application, which adds a layer of control as to who can see data and can prevent changes being made. In the medium to long term adding full support for row-level security is a planned development, but care must be taken that this does not adversely impact usability or performance too significantly.

Additional forthcoming features include support for hierarchical collections of objects such as clusters of genes or results, additional import and export options, more sophisticated database visualization tools and, eventually, a fully configurable schema. We will also be further developing the web-services interface to the maxd databases through maxdBrowse to allow incorporation of microarray data into the bioinformatics workflows supported through the Taverna component of ^my^Grid 18. This should make it much more straightforward for users to develop complex queries combining microarray data with other resources (such as Ensmart 19).

## Conclusion

maxdLoad2 is a free, highly-tested standards-compliant, infinitely configurable, post-genomic experimental annotation tool, presently directed at transcriptomics array based experiments. Developed to the needs of lab based experimentalists, it can act as a pipeline for annotation into public repositories such as ArrayExpress. maxd databases can be disseminated over the internet using maxdBrowse, either in a human-friendly manner to users through a web browser, or via web-services or command-line scripts into popular analysis packages. This allows both local and remote users to browse and download data, providing a security model for dissemination of private datasets to collaborators. maxdBrowse can be implemented either standalone or can be implemented as part of maxd-based microarray data repositories and can provide a PHP library for more complex web-based maxd-related queries in the future such as search, filtering and data processing.

## Availability and requirements

Project name: maxdLoad2

Project home page: 

Operating system(s): Platform independent

Programming language: Java

Other requirements: e.g. Java (1.4 or higher recommended), access to SQL database

License: Perl Artistic

Any restrictions to use by non-academics: None

Project name: maxdBrowse

Project home page: 

Operating system(s): Platform independent

Programming language: PHP 5

Other requirements: e.g. Web server (tested with Apache 2, IIS5), access to SQL database

License: Artistic

Any restrictions to use by non-academics: None

## List of abbreviations

API – Application Programmer Interface

MAGE-ML – Microarray and Gene Expression Markup Language

MAGE-OM – Microarray and Gene Expression Object Model

MGED – Microarray Gene Expression Data (Society) 

MO – MGED Ontology

NERC – Natural Environment Research Council

RDBMS – Relational Database Management System

SQL – Structured Query Language

XML – Extensible Markup Language 

## Authors' contributions

DH wrote the maxdLoad2 software and documentation; MW wrote the manuscript; GV wrote the maxdBrowse software and documentation; NM was responsible for development planning, schema design and testing maxdLoad2; AH was involved in design and testing of maxdLoad and providing ongoing use cases; HH was involved in feature design and testing of maxdLoad2 and training users; AJW was involved in feature design and testing of maxdLoad2/maxdBrowse, training of users, promoting the software and providing use cases; KN designed the usability studies and analysed the results; DK supervised, promoted and coordinated the development of maxdBrowse; AB supervised, promoted and coordinated the development of maxdLoad2. All authors read and approved the final manuscript.
